# When imitation meets exploration: quantifying social efficiency deficits (SED) in vaccination behaviour

**DOI:** 10.1098/rsif.2026.0682

**Published:** 2026-10-14

**Authors:** Akhil Kumar Srivastav, Chen Shen, Jun Tanimoto

**Affiliations:** ^1^ Interdisciplinary Graduate School of Engineering Sciences, Kyushu University, Kasuga, Fukuoka, Japan; ^2^ Faculty of Engineering Sciences, Kyushu University, Kasuga, Fukuoka, Japan

**Keywords:** vaccination behaviour, social dilemma, epidemic control

## Abstract

Vaccination behaviour plays a critical role in shaping epidemic dynamics, yet the societal consequences of decentralized vaccination decisions remain difficult to quantify. We develop a behavioural–epidemiological framework that combines adaptive vaccination dynamics, behavioural exploration and socially desirable vaccination strategies. To evaluate the societal impact of vaccination behaviour, we introduce the *social efficiency deficit* (SED), a novel metric that quantifies the welfare loss arising from decentralized vaccination decisions and measures the gap between individual incentives and collective epidemic-control objectives. Numerical simulations and global sensitivity analysis reveal that transmission intensity, waning immunity, vaccination costs, compliance and behavioural exploration are key determinants of epidemic outcomes and social welfare. In particular, behavioural exploration promotes sustained vaccination uptake, suppresses infection prevalence and can reduce social inefficiency by bringing behavioural outcomes closer to socially desirable levels. The proposed framework provides a quantitative approach for assessing vaccination-related social dilemmas and offers new insights for designing effective and socially efficient public health interventions.

## 1. Introduction

Vaccination is one of the most effective public health interventions for preventing infectious diseases and reducing epidemic burden. Immunization has played a central role in controlling diseases such as measles, polio, influenza, COVID-19 and Ebola, while providing both individual protection and population-level benefits through herd immunity [1,2]. However, the success of vaccination programmes ultimately depends on human behaviour, as vaccine uptake is influenced by perceived risks, costs, social influence, misinformation and individual decision-making [3,4]. The COVID-19 pandemic further demonstrated that behavioural responses, including vaccine hesitancy and risk perception, can substantially alter epidemic trajectories and public health outcomes [5,6]. Consequently, understanding the feedback between disease transmission and vaccination behaviour has become a central challenge in epidemiological modelling [7].

To address this challenge, behavioural epidemiology has increasingly incorporated concepts from vaccination behavioural epidemiological imitation dynamics and optimal control theory. Evolutionary vaccination games model vaccination decisions as strategic interactions in which individuals balance vaccination costs against infection risks and adapt their behaviour through imitation, learning or social influence. Previous studies have shown that imitation dynamics can generate complex vaccination patterns and significantly affect disease prevalence and control effectiveness. Game-theoretic approaches have been successfully applied to investigate voluntary vaccination, social distancing and behavioural responses during epidemics, revealing important discrepancies between individual incentives and collective welfare [8–18]. In parallel, optimal control theory has emerged as a powerful framework for determining intervention strategies that minimize epidemic burden while accounting for economic and logistical constraints. Numerous studies have employed Pontryagin's maximum principle to derive optimal vaccination, treatment, quarantine and transmission-reduction policies for epidemic systems [19–25]. Recent studies have begun to compare self-interested behavioural responses with socially optimal intervention strategies using integrated game-theoretic and optimal-control frameworks [26].

Other recent work has further emphasized that the emergence of cooperative behaviour does not necessarily coincide with the maximization of social welfare, highlighting the importance of explicitly distinguishing behavioural outcomes from socially desirable outcomes [27].

However, a quantitative measure of the welfare gap between decentralized behavioural outcomes and socially optimal epidemic policies remains largely unexplored. The social efficiency deficit (SED) introduced in this study addresses this limitation by directly quantifying the societal cost of behavioural self-interest, which directly compares Nash equilibrium (NE) vaccination behaviour with the corresponding socially optimal solution. Although both approaches have provided valuable insights, they are often developed independently: evolutionary game models focus on decentralized behavioural adaptation, whereas optimal control models typically assume centrally coordinated interventions. As a result, the relationship between individual behavioural decisions and socially optimal epidemic management remains insufficiently understood.

Motivated by this gap, we develop a behavioural–epidemiological framework that combines adaptive vaccination behaviour with socially optimal epidemic control. Vaccination decisions evolve through imitation dynamics and a behavioural exploration mechanism that captures spontaneous strategy switching arising from uncertainty and imperfect rationality. To evaluate the societal consequences of vaccination behaviour, we formulate an optimal vaccination control problem and introduce the SED, which quantifies the welfare gap between decentralized behavioural outcomes and socially optimal intervention policies.

The main contributions of this study are (i) the development of a coupled behavioural–epidemiological model; (ii) the introduction of a behavioural exploration mechanism; (iii) the derivation of a socially optimal vaccination policy; (iv) the adaptation of the SED framework [28–31] to vaccination behaviour; and (v) a systematic investigation of epidemiological, behavioural and economic drivers of social efficiency.

The proposed framework provides a quantitative approach for assessing vaccination-related social dilemmas and identifying conditions under which individual incentives diverge from collective public health objectives.

## 2. Method

### 2.1. Conceptual framework: behavioural exploration, optimal control and social efficiency deficit

Figure 1 summarizes the proposed framework, which combines adaptive vaccination behaviour, epidemic transmission and socially optimal intervention policies. A key innovation is the behavioural exploration term $\eta (1-2x_{V})$, which captures spontaneous strategy switching arising from uncertainty and imperfect information. Unlike conventional imitation-based models, this mechanism promotes persistent behavioural heterogeneity and prevents convergence to homogeneous vaccination states.

**Figure 1.**
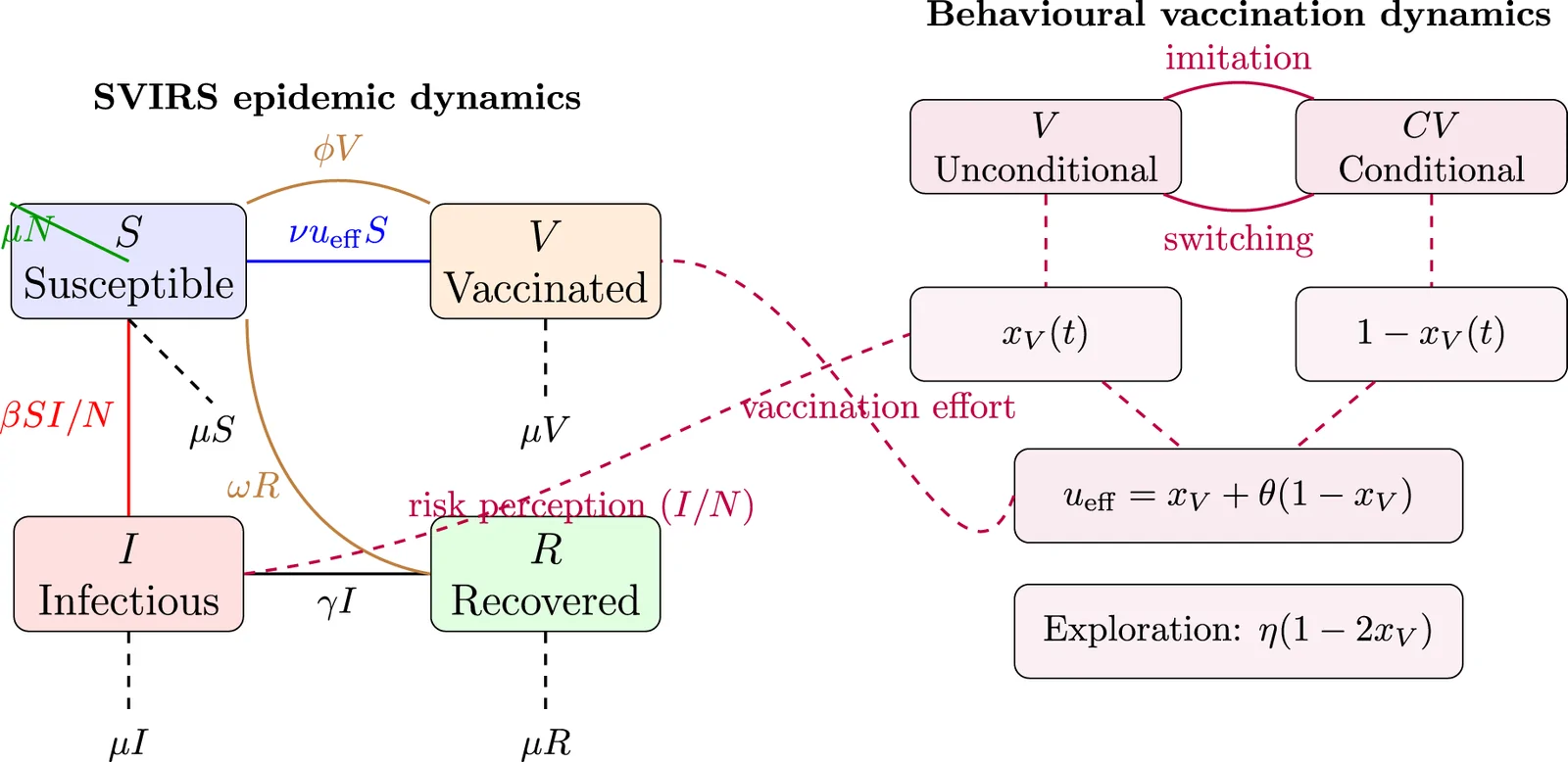
Schematic representation of the coupled behavioural–epidemiological SVIRS model. The behavioural module determines the effective vaccination effort $u_{\text{eff}}$, which drives vaccination from S to V. Vaccinated individuals return to susceptibility through waning vaccine-induced immunity at rate ϕV, while recovered individuals return to susceptibility through waning natural immunity at rate ωR.

Vaccination behaviour is coupled to an epidemic model, allowing disease prevalence and vaccine uptake to co-evolve. To evaluate socially desirable interventions, we formulate an optimal vaccination control problem that minimizes the overall societal burden associated with infection and vaccination costs.

A second contribution is the introduction of the SED, which quantifies the welfare loss associated with decentralized vaccination behaviour relative to the social optimum. Together, these components provide a framework for investigating how behavioural adaptation influences epidemic outcomes, vaccination dynamics and public health efficiency.

### 2.2. Model

#### 2.2.1. Behavioural decision-making and exploration

We consider a population in which individuals adopt one of two vaccination strategies: unconditional vaccination (V) and conditional vaccination (CV). Let $x_{V}(t)$ denote the proportion of unconditional vaccinators, while $1-x_{V}(t)$ represents conditional vaccinators.

Vaccination behaviour evolves through social imitation based on pay-off comparison [8,9]. The classical replicator dynamics is as follows:

(2.1) $$\frac{dx_{V}}{dt}=\kappa x_{V}(1-x_{V})(\Pi_{V}-\Pi_{CV}),$$

where κ>0 denotes the imitation rate and $\Pi_{V}$ and $\Pi_{CV}$ are the expected pay-offs associated with the two vaccination strategies.

To capture behavioural uncertainty, misinformation, experimentation and spontaneous reconsideration of vaccination decisions, we introduce a behavioural exploration mechanism. The resulting behavioural dynamics becomes

(2.2) $$\frac{dx_{V}}{dt}=\kappa x_{V}(1-x_{V})(\Pi_{V}-\Pi_{CV})+\eta (1-2x_{V}).$$

The exploration term $\eta (1-2x_{V})$ represents symmetric, pay-off-independent switching between the two vaccination strategies, analogous to mutation or spontaneous strategy switching in replicator–mutator dynamics [32]. Specifically, exploration-driven switching into the unconditional vaccination strategy occurs at rate $\eta (1-x_{V})$, whereas switching away from it occurs at rate $\eta x_{V}$. The resulting net exploration flow is, therefore, $\eta (1-x_{V})-\eta x_{V}=\eta (1-2x_{V})$. Thus, the factor $2x_{V}$ arises naturally from assuming equal exploration rates in both directions. Under this symmetric assumption, $x_{V}=0.5$ is the neutral point of the exploration process, while exploration tends to prevent complete behavioural convergence towards either strategy, where η≥0 denotes the exploration rate. The first term represents pay-off-driven social learning, whereas the second term describes spontaneous strategy switching independent of pay-off comparison. This mechanism prevents complete behavioural convergence and allows persistent coexistence of competing vaccination attitudes. The incorporation of behavioural exploration constitutes a central novelty of the present study.

The pay-off associated with unconditional vaccination is

(2.3) $$\Pi_{V}=-C_{V},\;\;\Pi_{CV}=-p\;\left(\frac{I}{N}\right)C_{V}-\left(1-p\;\left(\frac{I}{N}\right)\right)C_{I}\frac{I}{N},$$

where $C_{V}$ denotes the perceived vaccination cost. Conditional vaccinators evaluate vaccination according to epidemic prevalence and $C_{I}$ denotes the perceived infection cost. Following standard behavioural epidemic models, we assume

(2.4) $$p\;\left(\frac{I}{N}\right)=\frac{I}{N},$$

so that willingness to vaccinate increases with disease prevalence.

#### 2.2.2. Coupled behavioural–epidemiological dynamics

Behavioural adaptation and epidemic transmission are coupled through vaccination uptake. The effective vaccination effort is defined as

(2.5) $$u_{\text{eff}}(t)=x_{V}(t)+\theta (1-x_{V}(t)),$$

where $x_{V}(t)$ denotes the proportion of unconditional vaccinators and θ∈[0,1] represents the compliance level among conditional vaccinators. Here, θ=1 corresponds to full compliance and θ=0 to complete refusal.

The population is partitioned into susceptible (S), vaccinated (V), infectious (I) and recovered (R) individuals satisfying

$$\begin{array}{r} N(t)=S(t)+V(t)+I(t)+R(t). \end{array}$$

The coupled behavioural–epidemiological system is given by

(2.6)dSdt=μN−βSIN−νueffS−μS+ωR+ϕV,(2.7)dVdt=νueffS−(ϕ+μ)V,(2.8)dIdt=βSIN−(γ+μ)I,(2.9)dRdt=γI−(ω+μ)R,(2.10)dxVdt=κxV(1−xV)(ΠV−ΠCV)+η(1−2xV).

Here, μ denotes the birth-death rate, β the transmission rate, γ the recovery rate, ω the waning rate of natural immunity, ϕ the waning rate of vaccine-induced immunity, ν the baseline vaccination rate, κ the imitation rate and η the behavioural exploration rate. The total population is conserved and normalized such that N(t)=1.

The first four equations describe disease transmission and immunity dynamics, whereas the final equation governs vaccination behaviour through pay-off-driven imitation and spontaneous strategy switching. Together, these equations capture the feedback between epidemic prevalence and vaccination decisions.

### 2.3. Epidemiological and social performance measures

To quantify the societal impact of vaccination behaviour, we next define cumulative vaccination uptake, cumulative infection burden and average social pay-off (ASP) under both decentralized behavioural dynamics and socially optimal vaccination strategies. These measures form the basis of the SED, which compares behavioural and socially desirable outcomes within a common framework. The corresponding formulations of ${\text{ASP}}_{\text{NE}}$ and ${\text{ASP}}_{\text{SO}}$ are presented below.

#### 2.3.1. Average social pay-off under Nash equilibrium

Under decentralized behavioural dynamics, vaccination uptake is determined by the effective vaccination effort

(2.11) $$u_{\text{NE}}(t)=x_{V}(t)+\theta (1-x_{V}(t)),$$

which emerges from the coupled behavioural–epidemiological system.

*Cumulative vaccination uptake*. The total number of vaccinations administered over the time horizon [0,T] is

(2.12) $${\text{VT}}_{\text{NE}}=\int_{0}^{T}\nu u_{\text{NE}}(t)S(t)\;dt.$$

*Cumulative infection burden*. The cumulative incidence of infection is given by

(2.13) $${\text{IT}}_{\text{NE}}=\int_{0}^{T}\beta S(t)I(t)\;dt,$$

where the normalization N(t)=1 has been used.

*Average social pay-off*. To evaluate the societal consequences of decentralized vaccination behaviour, we define the instantaneous societal burden as

(2.14) $$Q_{\text{NE}}(t)=C_{I}\beta S(t)I(t)+C_{V}\nu u_{\text{NE}}(t)S(t),$$

where $C_{I}$ and $C_{V}$ denote the costs associated with infection and vaccination, respectively.

The cumulative societal burden generated under NE behaviour is

(2.15) $$J_{\text{NE}}=\int_{0}^{T}Q_{\text{NE}}^{2}(t)\;dt.$$

Substituting equation (2.14) into equation (2.15) yields

(2.16) $$J_{\text{NE}}=\int_{0}^{T}{\left[C_{I}\beta S(t)I(t)+C_{V}\nu (x_{V}(t)+\theta (1-x_{V}(t)))S(t)\right]}^{2}dt.$$

The ASP under decentralized behavioural dynamics is then defined as

(2.17) $${\text{ASP}}_{\text{NE}}=-\frac{1}{T}J_{\text{NE}}.$$

Equivalently,

(2.18) $${\text{ASP}}_{\text{NE}}=-\frac{1}{T}\int_{0}^{T}{\left[C_{I}\beta S(t)I(t)+C_{V}\nu (x_{V}(t)+\theta (1-x_{V}(t)))S(t)\right]}^{2}dt.$$

Since ${\text{ASP}}_{\text{NE}}$ is defined as the negative average societal burden, larger values correspond to lower overall burden and therefore more desirable decentralized outcomes.

#### 2.3.2. Socially optimal vaccination strategy

To evaluate socially desirable vaccination policies, we formulate an optimal control problem in which a central planner seeks to minimize the overall societal burden associated with infection transmission and vaccination interventions. By contrast to the decentralized behavioural dynamics, vaccination effort is represented by a time-dependent control variable

$$\begin{array}{r} u(t)\in [0,1], \end{array}$$

which denotes the proportion of susceptible individuals vaccinated at time t.

The controlled epidemiological system is

(2.19)dSdt=μN−βSI−νuS−μS+ωR+ϕV,(2.20)dVdt=νuS−(ϕ+μ)V,(2.21)dIdt=βSI−(γ+μ)I,(2.22)dRdt=γI−(ω+μ)R,

where the total population is normalized such that N(t)=1.

The admissible control set is

$$\begin{array}{r} U=\left\{u(\cdot )\in L^{\infty }(0,T):0\leq u(t)\leq 1\right\}. \end{array}$$

### 2.4. Objective functional

Unlike conventional epidemic optimal-control formulations, where the quadratic term is typically applied only to the control effort, we apply the quadratic form to the total instantaneous societal burden,

$$\begin{array}{r} Q(t)=C_{I}\beta S(t)I(t)+C_{V}\nu u(t)S(t). \end{array}$$

This choice is made to maintain consistency between the optimal-control objective and the ASP used in the SED framework. Squaring the combined burden introduces an interaction term between infection- and vaccination-related costs, thereby penalizing situations in which substantial infection burden and vaccination expenditure occur simultaneously. Thus, the objective evaluates their combined societal impact rather than treating the two cost components independently.

The instantaneous societal burden is defined as

(2.23) $$Q(t)=C_{I}\beta S(t)I(t)+C_{V}\nu u(t)S(t),$$

where $C_{I}$ and $C_{V}$ represent the costs associated with infection and vaccination, respectively.

The social planner seeks to minimize the cumulative burden

(2.24) $$J(u)=\int_{0}^{T}{\left[C_{I}\beta S(t)I(t)+C_{V}\nu u(t)S(t)\right]}^{2}dt.$$

The socially optimal vaccination policy is, therefore,

$$\begin{array}{r} u^{*}(t)=\arg\min_{u\in U}J(u), \end{array}$$

subject to the controlled state equations.

### 2.5. Optimality conditions

Applying Pontryagin's maximum principle, the Hamiltonian is

(2.25) $$\begin{array}{rl} H & ={\left[C_{I}\beta SI+C_{V}\nu uS\right]}^{2}\\ & \;+\lambda_{1}\left(\mu -\beta SI-\nu uS-\mu S+\omega R+\phi V\right)\\ & \;+\lambda_{2}\left(\nu uS-(\phi +\mu )V\right)\\ & \;+\lambda_{3}\left(\beta SI-(\gamma +\mu )I\right)\\ & \;+\lambda_{4}\left(\gamma I-(\omega +\mu )R\right). \end{array}$$

The adjoint variables satisfy

$$\begin{array}{r} \frac{d\lambda_{i}}{dt}=-\frac{\partial H}{\partial x_{i}},\;i=1,\ldots ,4, \end{array}$$

with transversality conditions

$$\begin{array}{r} \lambda_{i}(T)=0,\;i=1,\ldots ,4. \end{array}$$

The resulting adjoint equations are

(2.26)λ˙1=−[2Q(CIβI+CVνu)+λ1(−βI−νu−μ)+λ2νu+λ3βI],(2.27)λ˙2=−[λ1ϕ−λ2(ϕ+μ)],(2.28)λ˙3=−[2Q(CIβS)+λ1(−βS)+λ3(βS−(γ+μ))+λ4γ],(2.29)λ˙4=−[λ1ω−λ4(ω+μ)].

The optimal control is characterized by the stationarity condition

$$\begin{array}{r} \frac{\partial H}{\partial u}=0, \end{array}$$

which yields

(2.30) $$u^{*}(t)=\min\left\{1,\max\left[0,\frac{\lambda_{1}-\lambda_{2}-2C_{I}C_{V}\beta SI}{2C_{V}^{2}\nu S}\right]\right\}.$$

The existence of an optimal control follows from the Fleming–Rishel standard results, since the admissible control set is convex and closed, the state system is well posed, and the objective functional is continuous and convex with respect to the control variable.

#### 2.5.1. Epidemiological outcomes under the social optimum

The socially optimal vaccination policy $u^{*}(t)$ obtained from the optimality system allows the computation of cumulative vaccination uptake, infection burden and social welfare over the planning horizon [0,T].

*Cumulative vaccination uptake*. The total number of vaccinated individuals under the socially optimal policy is

(2.31) $${\text{VT}}_{\text{SO}}=\int_{0}^{T}\nu u^{*}(t)S(t)\;dt.$$

*Cumulative infection burden*. The cumulative infection burden is given by

(2.32) $${\text{IT}}_{\text{SO}}=\int_{0}^{T}\beta S(t)I(t)\;dt.$$

*Average social pay-off*. Since the objective functional J(u) represents the cumulative societal burden, the ASP under the socially optimal vaccination policy is defined as

(2.33) $${\text{ASP}}_{\text{SO}}=-\frac{1}{T}J(u^{*})=-\frac{1}{T}\int_{0}^{T}{\left[C_{I}\beta S(t)I(t)+C_{V}\nu u^{*}(t)S(t)\right]}^{2}dt.$$

Because ${\text{ASP}}_{\text{SO}}$ is defined as the negative average societal burden, larger values correspond to lower overall burden and, therefore, more desirable epidemic outcomes.

#### 2.5.2. Social efficiency deficit

Behavioural vaccination models describe how vaccination decisions emerge from individual incentives, whereas epidemic-control approaches identify vaccination strategies that improve population-level outcomes. These perspectives may lead to different epidemic trajectories because individual decisions do not always align with collective welfare. To quantify this discrepancy, we introduce the SED [28–31], which measures the welfare loss associated with decentralized vaccination behaviour relative to socially desirable outcomes.

The SED is defined as

(2.34) $$\text{SED}={\text{ASP}}_{\text{SO}}-{\text{ASP}}_{\text{NE}},$$

where ${\text{ASP}}_{\text{SO}}$ and ${\text{ASP}}_{\text{NE}}$ denote the ASP under the socially optimal vaccination strategy and the behavioural vaccination dynamics, respectively.

Since larger social pay-offs correspond to lower societal burden, a positive SED indicates that decentralized vaccination behaviour is less efficient than the socially desirable strategy. Larger values of SED, therefore, reflect a stronger vaccination-related social dilemma and a greater potential benefit from coordinated public health interventions, whereas values close to zero indicate close agreement between individual behaviour and collective objectives.

The SED thus provides a quantitative framework for evaluating the efficiency of vaccination behaviour and assessing the gap between individual incentives and population-level epidemic control.

### 2.6. Parameter values and description

The epidemiological parameters were chosen to represent a generic vaccine-preventable infectious disease and are consistent with values commonly used in epidemic modelling studies [21,22,26]. Behavioural parameters governing imitation, compliance and exploration were selected within plausible ranges motivated by previous vaccination game-theoretic models [3,8,9,17,28]. The cost parameters $C_{I}$ and $C_{V}$ are dimensionless quantities representing perceived infection and vaccination burdens and were normalized following standard practice in behavioural epidemiology. Since the primary objective is to investigate qualitative behavioural and epidemiological mechanisms, extensive sensitivity analyses were performed over broad parameter ranges.

## 3. Results

### 3.1. Numerical methods and social efficiency deficit

Numerical simulations were performed in MATLAB [33] to compare decentralized behavioural vaccination dynamics with socially optimal vaccination policies over a finite time horizon. The behavioural model was solved under NE dynamics, while the socially optimal vaccination strategy was obtained using an optimal-control framework (backward–forward sweep method) [34] based on Pontryagin's maximum principle [19]. Full details of the numerical algorithms, discretization procedures and optimal-control implementation are provided in the supplementary material.

### 3.2. Global sensitivity analysis

To evaluate the robustness of the model predictions, a global sensitivity analysis was performed using Latin hypercube sampling (LHS) combined with partial rank correlation coefficients (PRCC) [35,36]. Parameter values were sampled uniformly over biologically plausible ranges (see table 1), and the resulting effects on the SED were quantified. PRCC values were subsequently computed to identify the parameters exerting the strongest influence on social inefficiency. Positive PRCC values indicate that increasing a parameter increases SED, whereas negative values indicate that increasing a parameter reduces SED.

**Table 1.** Baseline parameter values and ranges considered in sensitivity analyses.

parameter	description	baseline	range
μ	natural birth/death rate	0.01	—
β	transmission rate	1.5	[0.2,3.0]
ν	vaccination rate	0.4	[0.05,1.0]
γ	recovery rate	0.2	—
ω	waning natural immunity	0.05	—
ϕ	waning vaccine immunity	0.03	[0.001,0.2]
κ	imitation rate	2.0	—
θ	compliance level	0.5	[0,1]
η	behavioural exploration rate	0.01	[0,0.1]
$C_{I}$	infection cost	1.0	[0.1,2.0]
$C_{V}$	vaccination cost	0.2	[0.05,1.0]

Figure 2 presents the results of the global sensitivity analysis based on LHS and PRCC for the ASP, cumulative infection burden, and cumulative vaccination uptake under both NE and socially optimal vaccination strategies. Overall, the vaccination rate (ν), transmission rate (β), vaccine waning rate (ϕ), compliance level (θ) and behavioural exploration rate (η) emerge as the most influential parameters, indicating that both epidemiological and behavioural factors play important roles in determining epidemic outcomes and social welfare.

**Figure 2.**
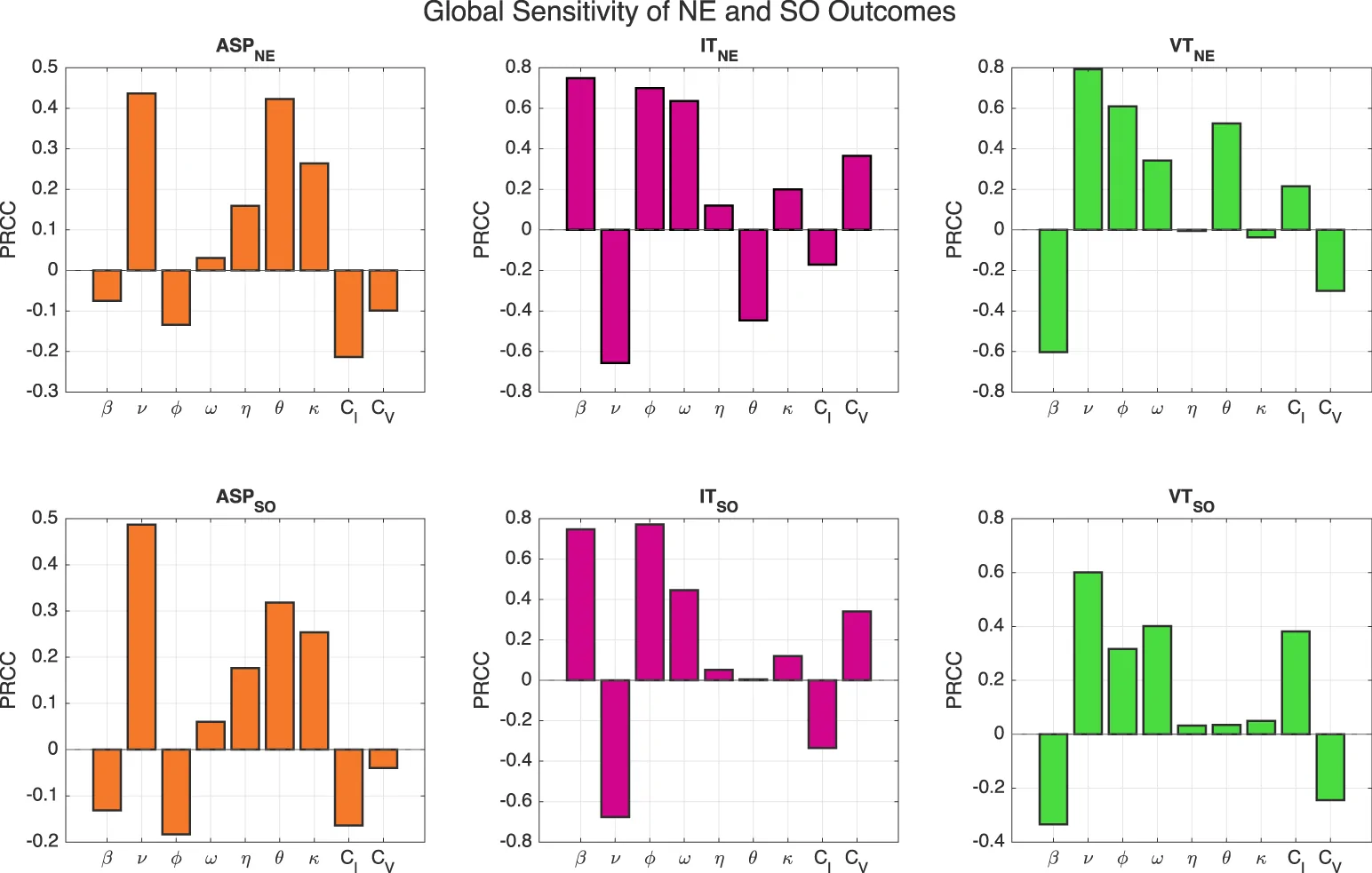
Global sensitivity analysis of epidemiological and social outcomes under Nash equilibrium (NE) and socially optimal (SO) vaccination strategies. PRCC values are shown for the ASP, cumulative infection burden and cumulative vaccination uptake; positive and negative PRCC values indicate parameters that increase or decrease the corresponding outcome, respectively. The results illustrate the relative influence of epidemiological, behavioural and economic parameters on both decentralized and socially coordinated vaccination dynamics.

For the cumulative infection burden (IT), the transmission rate (β), vaccine waning rate (ϕ), natural immunity waning rate (ω) and vaccination cost ($C_{V}$) exhibit positive PRCC values, indicating that increases in these parameters lead to higher infection burden. By contrast, the vaccination rate (ν) has the strongest negative influence, confirming that increased vaccination coverage is the most effective mechanism for reducing disease transmission. Under the behavioural framework (NE), compliance (θ) and behavioural exploration (η) also contribute substantially to lowering infection burden, whereas their effects are less pronounced under the socially optimal strategy.

The vaccination uptake results (VT) reveal that higher vaccination rates and stronger compliance substantially increase cumulative vaccination coverage. Under NE, behavioural exploration has a noticeable positive effect on vaccination uptake, reflecting its role in maintaining a persistent fraction of vaccinators within the population. This influence is considerably weaker under social optimality because vaccination effort is determined directly by the socially optimal intervention policy rather than by individual behavioural adaptation.

A similar pattern is observed for the ASP. Parameters that promote vaccination and reduce infections, particularly ν, θ and η, generally improve social welfare, whereas faster loss of immunity (ϕ) and higher vaccination costs ($C_{V}$) reduce societal benefit. Notably, behavioural exploration exerts a stronger influence on ${\text{ASP}}_{NE}$ than on ${\text{ASP}}_{SO}$, suggesting that behavioural variability can improve collective outcomes by preventing the system from becoming trapped in low-vaccination states.

Taken together, these results demonstrate that the model predictions are robust to parameter uncertainty and highlight the important role of behavioural processes in shaping epidemic outcomes. The stronger sensitivity of NE outcomes to behavioural parameters further supports the central premise of this study that behavioural adaptation is a key determinant of vaccination dynamics and the resulting social efficiency of epidemic-control strategies.

Figure 3 presents the global sensitivity analysis of the SED based on LHS and PRCC. The results identify the parameters that most strongly influence the welfare gap between decentralized vaccination behaviour and socially desirable epidemic-control outcomes.

**Figure 3.**
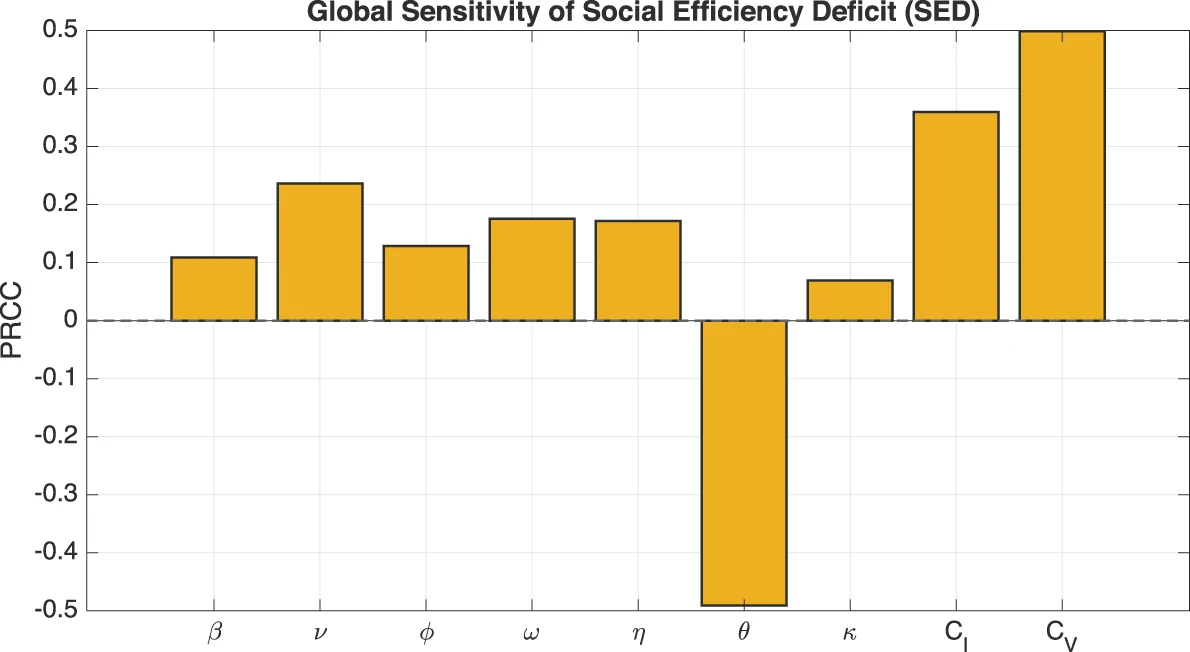
Global sensitivity analysis of the SED using LHS and PRCC. Positive PRCC values indicate parameters that increase social inefficiency, whereas negative values indicate parameters that reduce the welfare gap between decentralized vaccination behaviour and socially desirable vaccination outcomes. The analysis identifies the epidemiological, behavioural and economic factors that most strongly influence the discrepancy between individual incentives and collective welfare.

The strongest positive PRCC values are associated with the infection cost ($C_{I}$) and vaccination cost ($C_{V}$), indicating that economic factors exert the greatest influence on social inefficiency. Higher costs amplify the welfare consequences of discrepancies between individual vaccination decisions and socially coordinated strategies, thereby increasing SED. The vaccination rate (ν) also exhibits a positive influence on SED, suggesting that as vaccination becomes more widely available, differences between behavioural and socially optimal responses can become more pronounced. Similarly, faster waning of vaccine-induced immunity (ϕ) and natural immunity (ω) increase SED by reducing long-term protection and weakening the alignment between individual incentives and collective objectives.

By contrast, the compliance parameter (θ) displays the largest negative PRCC value, identifying it as the most effective mechanism for reducing social inefficiency. Greater compliance increases vaccination uptake among conditional vaccinators and brings decentralized behavioural outcomes closer to socially desirable vaccination levels. Behavioural exploration (η) and the imitation rate (κ) exert comparatively weaker influences, indicating that their effects on SED are secondary to those of compliance, immunity waning and cost-related factors. Although behavioural exploration increases vaccination uptake and reduces infection under the behavioural model, its global effect on SED is context-dependent. This is because η directly affects only the NE behavioural dynamics, whereas the socially optimal control does not depend explicitly on η. Since SED measures the difference between the socially optimal pay-off and the behavioural pay-off, its sensitivity to η depends on how exploration interacts with other parameters such as compliance, vaccination cost, transmission intensity and waning immunity. Therefore, a positive PRCC for η does not contradict the time-series results; rather, it indicates that the contribution of exploration to social inefficiency varies across the broader parameter space.

Overall, the analysis demonstrates that the SED is jointly shaped by epidemiological, behavioural and economic parameters. The dominant negative effect of compliance highlights the importance of sustained vaccine engagement in reducing the welfare gap between individual behaviour and collective public health objectives.

To further understand the mechanisms of SED, identified by the global sensitivity analysis, we performed detailed parameter-sweep investigations using two-parameter heatmaps together with time-series comparisons of behavioural (NE) and socially optimal vaccination dynamics.

### 3.3. Comparative analysis of behavioural and socially optimal dynamics: analysis of average social pay-off and social efficiency deficit

In this section, we investigate how epidemiological parameters, behavioural exploration, partial compliance and economic costs collectively influence vaccination dynamics and epidemic outcomes under both decentralized and socially optimal decision-making frameworks. Specifically, we compare the NE behavioural dynamics derived from imitation-based vaccination decisions with the socially optimal solutions obtained through optimal control theory. To quantify the resulting epidemiological and societal impacts, we analyse the cumulative vaccination uptake, cumulative infection burden, ASP and the SED across multiple parameter regimes. Particular emphasis is placed on understanding how random exploration and behavioural adaptation modify the discrepancy between individual and societal objectives, thereby shaping vaccination-related social dilemmas and epidemic control efficiency.

#### 3.3.1. Impact of behavioural exploration and partial compliance on social dilemmas

Figure 4 illustrates the combined effects of behavioural exploration (η) and partial compliance (θ) on vaccination uptake, infection burden, social pay-off and the SED. Under decentralized behavioural dynamics (NE), increasing compliance substantially increases cumulative vaccination uptake (${\text{VT}}_{\text{NE}}$) while reducing cumulative infections (${\text{IT}}_{\text{NE}}$), resulting in improved social outcomes (${\text{ASP}}_{\text{NE}}$). Behavioural exploration exerts an additional stabilizing influence by maintaining a persistent fraction of unconditional vaccinators, as reflected in the increase of the equilibrium vaccination level $x_{V}$. Consequently, higher exploration rates prevent convergence towards low-vaccination behavioural states and reduce epidemic burden even when compliance remains imperfect.

**Figure 4.**
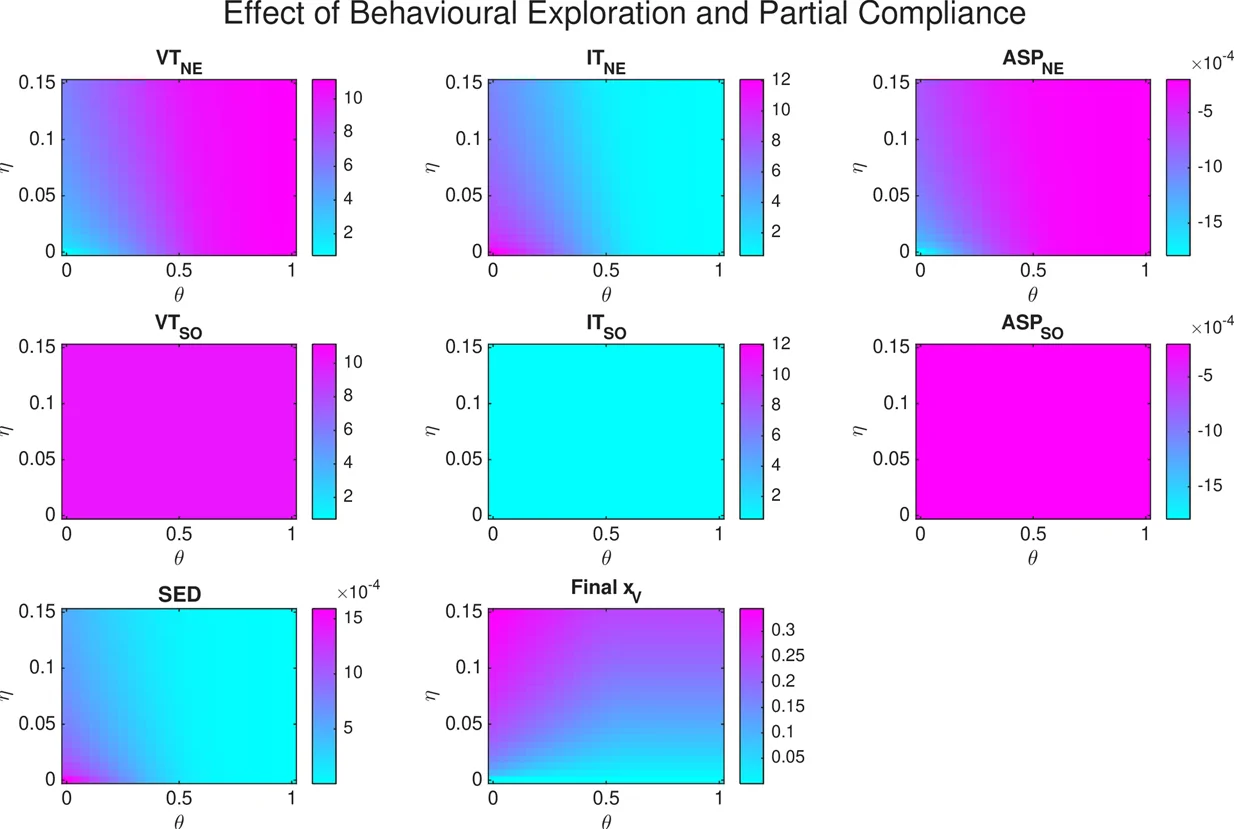
Effects of behavioural exploration (η) and partial compliance (θ) on epidemic outcomes, social welfare and the SED under NE and socially optimal (SO) vaccination strategies. The final panel shows equilibrium vaccination behaviour ($x_{V}$), illustrating the role of exploration in shaping long-term vaccination dynamics.

By contrast, the socially optimal solution is comparatively insensitive to η and θ, since vaccination effort is determined directly through the optimal control policy rather than through behavioural adaptation. As a result, vaccination uptake, infection burden and social pay-off remain relatively stable across the parameter space under the socially optimal strategy.

The most important result is observed in the SED. The SED reaches its largest values when both compliance and exploration are low, indicating a substantial discrepancy between decentralized behavioural decisions and the socially optimal vaccination policy. As compliance and behavioural exploration increase, vaccination behaviour moves closer to the socially desirable outcome, reducing the societal costs associated with decentralized decision-making.

This finding conveys a particularly important message. Random exploration is often viewed as a source of behavioural noise or mutation in evolutionary dynamics [28,37,38]. By contrast, our results suggest that behavioural exploration can improve collective epidemic outcomes by reducing social inefficiency and bringing vaccination behaviour closer to socially desirable levels. Thus, exploration is not merely a source of randomness; it acts as a mechanism that reduces social inefficiency and mitigates the welfare losses generated by purely pay-off-driven imitation dynamics. This observation represents a central novelty of the present study and highlights an unexpected pathway through which behavioural variability can enhance epidemic resilience.

From a public health perspective, these findings suggest that interventions promoting behavioural flexibility, information seeking, and continued engagement with vaccination programmes may improve epidemic outcomes even in the absence of strict mandates. Encouraging individuals to periodically reassess vaccination decisions may therefore help reduce vaccination-related social dilemmas and improve alignment between individual behaviour and collective welfare.

#### 3.3.2. Effect of vaccination rate and waning vaccine immunity

Figure 5 illustrates the combined effects of the vaccination rate (ν) and the waning rate of vaccine-induced immunity (ϕ) on vaccination uptake, infection burden, social pay-off and the SED. Under decentralized behavioural dynamics (NE), increasing the vaccination rate substantially increases cumulative vaccination uptake (${\text{VT}}_{\text{NE}}$) and reduces cumulative infections (${\text{IT}}_{\text{NE}}$), resulting in improved social outcomes (${\text{ASP}}_{\text{NE}}$). Conversely, faster waning of vaccine-induced immunity weakens long-term protection, leading to higher infection burden and lower social pay-off despite continued vaccination efforts. Similar qualitative trends are observed under the socially optimal strategy, although the optimal controller consistently achieves greater vaccination coverage and lower infection burden than the decentralized behavioural system.

**Figure 5.**
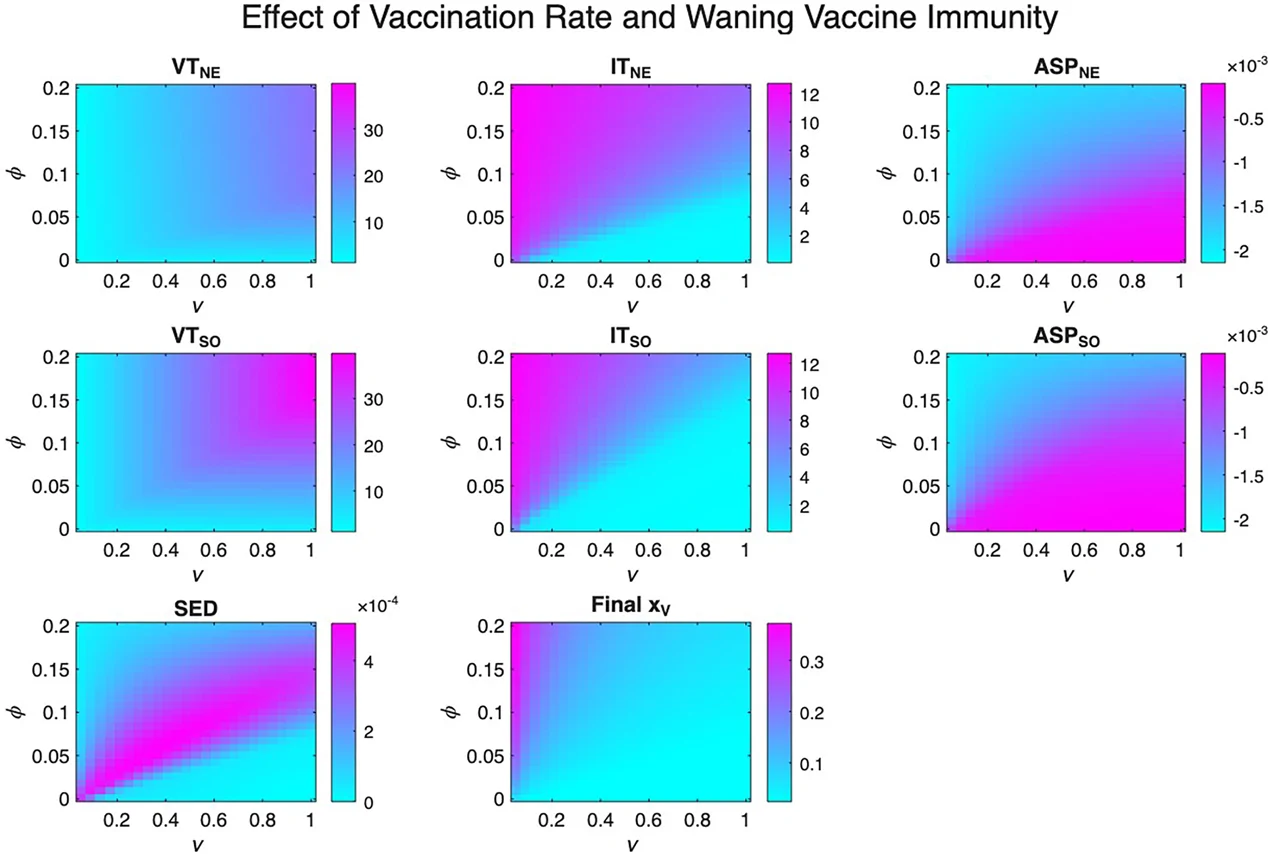
Heatmaps showing the effects of vaccination rate (ν) and waning vaccine-induced immunity (ϕ) on epidemic outcomes, social welfare, and the SED under NE and socially optimal (SO) vaccination strategies. The final panel shows the equilibrium proportion of unconditional vaccinators ($x_{V}$).

From an epidemiological perspective, these patterns are largely expected: higher vaccination rates and more durable immunity improve disease control. However, the most interesting result emerges from the SED. The SED is not maximized under the most adverse epidemiological conditions, but instead reaches its highest values within an intermediate region characterized by moderate vaccination rates and moderate levels of vaccine protection. In this regime, vaccination remains sufficiently beneficial to encourage uptake by part of the population, while simultaneously creating opportunities for others to rely on the indirect protection generated by vaccinated individuals. As a result, individual incentives become increasingly misaligned with collective welfare, leading to the largest discrepancy between decentralized behavioural outcomes and the socially optimal strategy.

This observation suggests that the strongest vaccination-related social dilemma may arise not when vaccination performs poorly, but when it performs well enough to generate substantial indirect protection while still allowing opportunities for free-riding. Consequently, moderate vaccine effectiveness can produce a larger welfare gap than either very weak or very strong protection. The socially optimal controller compensates for this behavioural inefficiency by maintaining vaccination levels that suppress transmission more effectively, thereby producing substantially better epidemic outcomes.

The final equilibrium vaccination panel further shows that stronger waning immunity generally reduces the proportion of unconditional vaccinators ($x_{V}$), indicating that declining vaccine effectiveness weakens long-term behavioural commitment to vaccination. From a public health perspective, these findings highlight the importance of maintaining durable vaccine protection through booster programmes and sustained vaccination campaigns. More broadly, the results demonstrate that epidemiological characteristics influence not only disease dynamics but also the magnitude of the underlying social dilemma, reinforcing the value of combining behavioural vaccination games with optimal-control approaches when designing vaccination policies.

#### 3.3.3. Effects of infection and vaccination costs on social efficiency

Figure 6 illustrates how the perceived infection cost ($C_{I}$) and vaccination cost ($C_{V}$) jointly influence vaccination uptake, infection burden, social welfare and the SED. As the perceived cost of infection increases, individuals become more willing to vaccinate, leading to higher cumulative vaccination uptake and lower infection burden under both behavioural and socially optimal strategies. By contrast, increasing the perceived vaccination cost discourages vaccine adoption, resulting in reduced vaccination coverage, increased disease transmission and lower social welfare. Although both approaches exhibit similar qualitative trends, the socially optimal strategy consistently achieves greater vaccination coverage and lower infection burden than the decentralized behavioural system.

**Figure 6.**
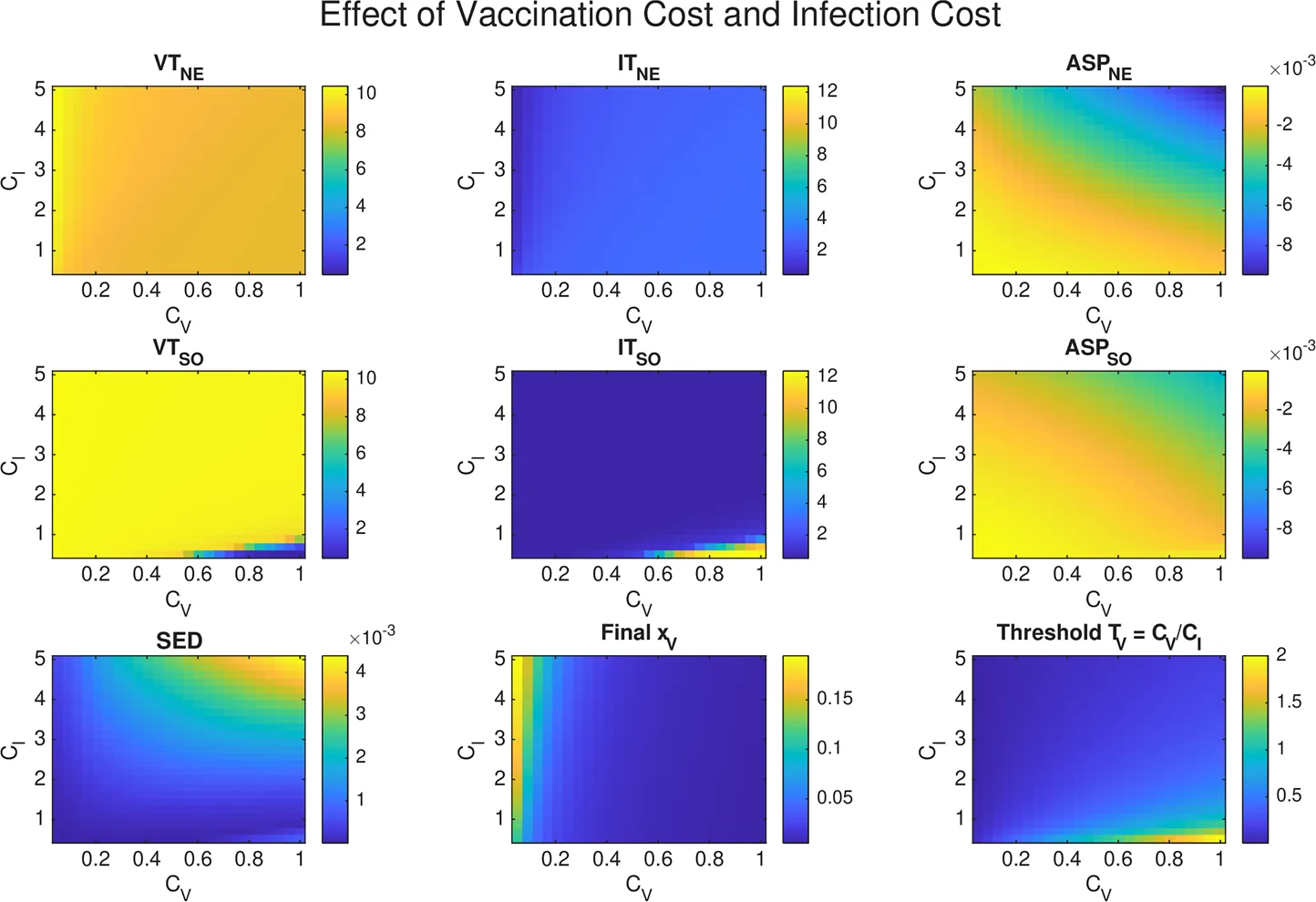
Impact of the infection cost ($C_{I}$) and vaccination cost ($C_{V}$) on vaccination uptake, infection burden, social welfare and the SED. Increasing infection costs generally promotes vaccination and improves epidemic outcomes, whereas increasing vaccination costs discourages vaccine uptake and increases disease burden. The largest values of SED occur when both costs are high, indicating a stronger conflict between individual incentives and socially desirable vaccination behaviour.

The most interesting pattern emerges in the SED landscape. Social inefficiency remains relatively low when either infection costs or vaccination costs are small, but increases substantially when both costs become large. In this region, individuals face competing incentives: vaccination is costly, yet avoiding infection is also increasingly important. As a consequence, decentralized behavioural responses fail to achieve the level of vaccination required to minimize the overall societal burden, whereas the socially optimal strategy maintains more effective epidemic control. The resulting divergence between individual and collective objectives produces larger values of SED and highlights a stronger vaccination-related social dilemma.

The equilibrium vaccination level further reveals that higher infection costs promote long-term commitment to vaccination, whereas higher vaccination costs reduce the fraction of persistent vaccinators. These results demonstrate that economic perceptions can strongly influence both epidemic outcomes and social efficiency. From a public health perspective, reducing the real or perceived costs of vaccination through affordable vaccination programmes, transparent communication and public health education may not only increase vaccine uptake but also reduce the welfare losses associated with decentralized decision-making.

### 3.4. Time-series comparison between decentralized behaviour and socially optimal control

Figure 7 illustrates the impact of behavioural exploration (η) (left-hand panel) on epidemic, behavioural and control dynamics under NE and socially optimal vaccination strategies. Increasing behavioural exploration reduces infection prevalence and increases vaccine coverage by maintaining a persistent fraction of vaccinators within the population. As shown in panel (c), when η=0, the proportion of unconditional vaccinators $x_{V}$ converges towards zero, whereas positive exploration sustains long-term behavioural diversity and prevents complete vaccination abandonment. This persistence of vaccination behaviour leads to lower endemic infection levels and improved epidemic outcomes under decentralized decision-making. By contrast, panel (d) shows that the socially optimal vaccination control $u^{*}(t)$ remains essentially unchanged across different values of η. This occurs because η influences only the behavioural game-theoretic dynamics governing $x_{V}(t)$ and does not appear in the optimal control formulation. Consequently, behavioural exploration modifies individual vaccination decisions and improves NE outcomes without altering the socially optimal intervention strategy itself. This result highlights a key contribution of the proposed framework: behavioural variability can substantially reduce epidemic burden and narrow the gap between decentralized and socially optimal outcomes, even though the optimal control benchmark remains unchanged.

**Figure 7.**
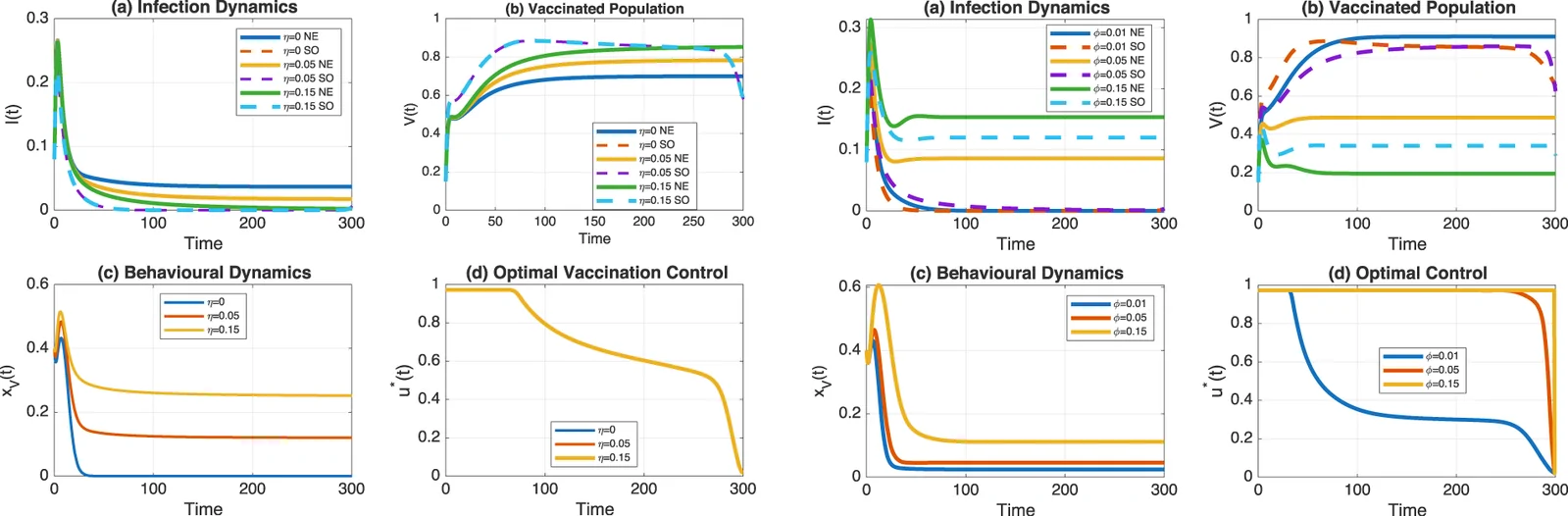
Time-series comparison of decentralized behavioural (NE) and socially optimal (SO) vaccination dynamics. Left-hand panels show the effects of behavioural exploration (η), while right-hand panels show the effects of waning vaccine-induced immunity (ϕ), on infection, vaccination, behavioural dynamics and optimal vaccination control.

Figure 7 illustrates that increasing ϕ (right-hand panel) substantially worsens epidemic outcomes by accelerating the loss of vaccine protection and continuously replenishing the susceptible population. As a result, infection prevalence remains higher and long-term vaccine coverage declines as immunity wanes more rapidly. Panel (c) shows that larger values of ϕ stimulate greater vaccination willingness, leading to higher levels of unconditional vaccination behaviour $x_{V}(t)$. This behavioural response reflects increased perceived infection risk in populations where vaccine protection is short-lived. Nevertheless, the increase in vaccination behaviour is insufficient to fully compensate for the epidemiological consequences of rapid immunity loss, and infection levels remain elevated. Panel (d) further demonstrates that the socially optimal vaccination control $u^{*}(t)$ becomes more aggressive as ϕ increases. When vaccine-induced immunity wanes rapidly, the social planner maintains high vaccination effort for a longer period to offset the continual return of vaccinated individuals to the susceptible class. The decline in the vaccinated population near the end of the time horizon is a consequence of the finite-horizon optimal-control solution. As t approaches the terminal time T, the optimal vaccination effort $u^{*}(t)$ decreases, reducing the vaccination inflow $\nu u^{*}S$. In addition, individuals continue to leave the vaccinated compartment at rate (ϕ+μ)V owing to waning vaccine-induced immunity and natural mortality. Consequently, the reduced inflow relative to the continuing outflow leads to the observed decline in V(t) near the end of the simulation period. These results highlight the critical public health importance of vaccine durability: although behavioural adaptation encourages additional vaccination under high waning rates, sustained and intensive vaccination programmes remain necessary to achieve effective epidemic control. The comparison between NE and socially optimal dynamics further emphasizes that coordinated intervention strategies become increasingly important as vaccine-induced immunity becomes less persistent.

Overall, the proposed framework links behavioural vaccination dynamics with socially optimal epidemic control through the SED, providing a quantitative measure of the welfare loss arising from decentralized decision-making. The results indicate that promoting adaptive vaccination behaviour can reduce social inefficiency and enhance epidemic control.

Note: Appendix A contains further mathematical details on existence of optimal control (appendix A.1), disease-free equilibrium (appendix A.1.1) and endemic equilibrium (appendix A.1.2).

## 4. Discussion and conclusion

### 4.1. Discussion

Vaccination behaviour inherently involves a social dilemma because the decision that is optimal for an individual may not coincide with what is best for the population as a whole. While vaccination generates collective benefits by reducing disease transmission, individuals may be reluctant to vaccinate because of perceived costs, risks or the possibility of relying on the protection provided by others. As a result, decentralized behavioural decisions can lead to vaccination levels that are insufficient from a public health perspective. Quantifying the extent of this discrepancy is therefore essential for understanding the societal consequences of voluntary vaccination. To address this issue, we introduce the SED, which measures the welfare loss associated with decentralized vaccination behaviour relative to socially desirable outcomes. The SED provides a direct indicator of the extent to which individual incentives diverge from collective objectives and identifies conditions under which vaccination-related social dilemmas become most severe.

Across all parameter regimes considered, socially optimal vaccination policies consistently outperformed decentralized behavioural dynamics, resulting in lower infection burden and improved social welfare. The largest values of SED were generally observed under conditions of high transmission intensity, rapid waning immunity and unfavourable cost structures. These findings indicate that the discrepancy between individual incentives and collective welfare becomes increasingly important when epidemiological risks are high or when maintaining vaccine-induced protection becomes more challenging.

The global sensitivity analysis further demonstrated that the principal conclusions of the study are robust to parameter uncertainty. Parameters associated with vaccination uptake and protection, particularly the vaccination rate, compliance level and immunity waning rates, exerted the strongest influence on epidemic outcomes and social efficiency. By contrast, higher transmission intensity and larger infection or vaccination costs generally increased the SED. These results are consistent with the heatmap and time-series analyses and provide additional evidence that both epidemiological and behavioural factors jointly determine the magnitude of vaccination-related social dilemmas.

An important finding of this study is that behavioural exploration can substantially reduce social inefficiency. Unlike conventional imitation dynamics, which often drives populations towards homogeneous behavioural states, exploration maintains persistent coexistence between competing vaccination attitudes by allowing spontaneous strategy switching independent of pay-off maximization. Consequently, higher exploration rates promote vaccination uptake, reduce infection burden and improve population-level welfare. More importantly, exploration systematically decreases the SED, indicating that decentralized behavioural outcomes move closer to socially desirable vaccination levels. This finding conveys a particularly important message: behavioural variability is often regarded as a source of noise in decision-making processes, yet our results suggest that exploration can instead improve collective epidemic outcomes by mitigating the inefficiencies associated with purely pay-off-driven behaviour. Rather than acting merely as randomness, behavioural exploration functions as a stabilizing mechanism that reduces welfare losses and narrows the gap between individual incentives and collective objectives. This highlights a previously underappreciated role of behavioural variability in promoting more effective epidemic control.

Another notable insight is that the largest social inefficiencies do not necessarily occur under the most adverse epidemiological conditions. Instead, the SED often reaches its highest values at intermediate levels of vaccine protection. In this regime, vaccination remains sufficiently beneficial to sustain vaccine uptake while simultaneously creating opportunities for some individuals to rely on the indirect protection generated by vaccinated members of the population. Consequently, the divergence between individual incentives and collective welfare becomes more pronounced, leading to larger welfare losses. This mechanism is consistent with vaccination dilemmas identified in previous game-theoretic studies [8,9,17,28], where free-riding behaviour emerges when individual and collective interests become misaligned.

From a public health perspective, these findings emphasize that effective epidemic management requires consideration of both epidemiological and behavioural processes. Policies that promote sustained vaccine engagement, behavioural flexibility and informed decision-making may help reduce the gap between individual behaviour and collective welfare. In practical terms, a small SED indicates that the societal burden generated by decentralized vaccination behaviour is close to that achieved under the socially optimal strategy, whereas a large SED indicates a substantial welfare loss arising from the misalignment between individual decisions and collective objectives. Because the absolute magnitude of SED depends on the scale of the underlying societal burden, its interpretation is most informative when considered relative to the corresponding social pay-off or societal burden and when comparing different epidemiological and behavioural scenarios. Thus, the SED framework provides a quantitative tool for identifying parameter regimes in which decentralized vaccination behaviour generates substantial social inefficiency and where coordinated interventions may provide the greatest societal benefit.

### 4.2. Conclusion

Understanding how individual vaccination decisions influence collective epidemic outcomes remains a major challenge for public health planning. In this study, we showed that behavioural adaptation, epidemiological processes and intervention policies interact in complex ways that can substantially affect both disease burden and societal welfare. In particular, behavioural exploration emerged as an important mechanism that promotes vaccination uptake, reduces infection burden and narrows the gap between decentralized behavioural outcomes and socially desirable vaccination policies.

A key contribution of this work is the introduction of the SED, which provides a quantitative measure of the welfare loss arising from voluntary vaccination behaviour. By identifying conditions under which individual incentives diverge most strongly from collective interests, the SED offers a useful framework for evaluating vaccination-related social dilemmas and assessing the effectiveness of public health interventions.

From a policy perspective, the results suggest that strategies promoting sustained vaccine engagement, behavioural flexibility and informed decision-making may improve epidemic outcomes even when centralized control is limited. More broadly, the proposed framework highlights the importance of incorporating behavioural processes into epidemic models and provides a foundation for designing vaccination policies that better align individual behaviour with population-level health objectives. The SED offers a practical quantitative tool for identifying conditions under which behavioural responses are most likely to undermine collective welfare and where targeted public health interventions may yield the greatest societal benefit.

## Data Availability

This article has no additional data.

## Appendix A

### A.1. Existence of an optimal control

Let the admissible control set be

(A 1) $$U=\left\{u(\cdot )\in L^{\infty }(0,T):0\leq u(t)\leq 1\;\text{for all}\;t\in [0,T]\right\}.$$

The objective functional is

(A 2) $$J(u)=\int_{0}^{T}{\left[C_{I}\beta S(t)I(t)+C_{V}\nu u(t)S(t)\right]}^{2}dt.$$

Theorem
There exists an optimal control

$$\begin{array}{r} u^{*}(t)\in U \end{array}$$

such that

$$\begin{array}{r} J(u^{*})=\min_{u\in U}J(u). \end{array}$$

Proof
The proof follows from the Fleming–Rishel classical existence theorem. First, the admissible control set U is non-empty, convex and closed in $L^{\infty }(0,T)$. Second, for every admissible control u∈U, the state system admits a unique solution because the right-hand sides of the state equations are continuously differentiable with respect to the state variables and linear in the control variable. Third, the state variables remain non-negative and bounded within the biologically feasible region

$$\begin{array}{r} \Omega =\left\{(S,V,I,R)\in R_{+}^{4}:S+V+I+R=1\right\}. \end{array}$$

Furthermore, the integrand

$$\begin{array}{r} L(S,I,u)={\left[C_{I}\beta SI+C_{V}\nu uS\right]}^{2} \end{array}$$

is continuous in both the state and control variables. Moreover,

$$\begin{array}{r} \frac{\partial^{2}L}{\partial u^{2}}=2(C_{V}\nu S{)}^{2}\geq 0, \end{array}$$

which implies convexity with respect to the control variable. Finally, the integrand satisfies a coercivity condition of the form

$$\begin{array}{r} L(S,I,u)\geq c_{1}u^{2}-c_{2}, \end{array}$$

for some constants $c_{1},c_{2}>0$. Therefore, all assumptions of the Fleming–Rishel existence theorem are satisfied. Consequently, there exists an optimal control

$$\begin{array}{r} u^{*}(t)\in U \end{array}$$

that minimizes the objective functional J(u). ◻

#### A.1.1. Disease-free equilibrium

The disease-free equilibrium (DFE) corresponds to the absence of infection in the population, that is,

$$\begin{array}{r} I^{0}=0,\;R^{0}=0. \end{array}$$

At equilibrium, the vaccinated compartment satisfies

(A 3) $$\nu (x_{V}^{0}+\theta (1-x_{V}^{0}))S^{0}=(\phi +\mu )V^{0},$$

which gives

(A 4) $$V^{0}=\frac{\nu (x_{V}^{0}+\theta (1-x_{V}^{0}))}{\phi +\mu }\;S^{0}.$$

Using the conservation relation $S^{0}+V^{0}=N$, we obtain

(A 5) $$S^{0}=\frac{N}{1+\frac{\nu (x_{V}^{0}+\theta (1-x_{V}^{0}))}{\phi +\mu }},$$

and consequently

(A 6) $$V^{0}=\frac{\nu (x_{V}^{0}+\theta (1-x_{V}^{0}))}{\phi +\mu }S^{0}.$$

Therefore, the DFE is

(A 7) $$E_{0}=\left(S^{0},V^{0},0,0\right).$$

The DFE depends explicitly on the effective vaccination effort $x_{V}^{0}+\theta (1-x_{V}^{0})$, indicating that behavioural compliance directly influences the level of residual susceptibility and the ability of the disease to invade the population.

#### A.1.2. Endemic equilibrium

The endemic equilibrium corresponds to a persistent infection state with

$$\begin{array}{r} I^{*}>0, \end{array}$$

and is obtained by setting

$$\begin{array}{r} \frac{dS}{dt}=\frac{dV}{dt}=\frac{dI}{dt}=\frac{dR}{dt}=\frac{dx_{V}}{dt}=0. \end{array}$$

The resulting equilibrium is

$$\begin{array}{r} E^{*}=(S^{*},V^{*},I^{*},R^{*},x_{V}^{*}), \end{array}$$

where

(A 8)S∗=N(γ+μ)β,(A 9)V∗=ν(xV∗+θ(1−xV∗))ϕ+μS∗,(A 10)I∗=N[1−γ+μβ(1+ν(xV∗+θ(1−xV∗))ϕ+μ)]1+γω+μ,(A 11)R∗=γω+μI∗.

The equilibrium vaccination level $x_{V}^{*}$ is determined by the behavioural equilibrium condition

(A 12) $$\kappa x_{V}^{*}(1-x_{V}^{*})(\Pi_{V}^{*}-\Pi_{CV}^{*})+\eta (1-2x_{V}^{*})=0.$$

When η=0, equation (A 12) reduces to the standard replicator equilibrium condition

$$\begin{array}{r} x_{V}^{*}=0,\;x_{V}^{*}=1, \end{array}$$

or an interior solution satisfying

$$\begin{array}{r} \Pi_{V}^{*}=\Pi_{CV}^{*}. \end{array}$$

For η≠0, the behavioural exploration term generally eliminates the boundary equilibria and promotes an interior equilibrium determined by the balance between pay-off-driven imitation and spontaneous strategy switching.

The endemic equilibrium therefore arises from a nonlinear feedback between epidemiological and behavioural dynamics. Disease prevalence influences vaccination behaviour through perceived infection risk, while vaccination behaviour simultaneously alters disease transmission through the effective vaccination effort. Consequently, epidemiological and behavioural equilibria must be determined simultaneously rather than independently.
